# Possible involvement of microglial P2RY12 and peripheral IL-10 in postpartum depression

**DOI:** 10.3389/fncel.2023.1162966

**Published:** 2023-06-15

**Authors:** Hui-Ju Kim, Min-Jung You, Soyoung Sung, Chan Rim, Min-Soo Kwon

**Affiliations:** Department of Pharmacology, Research Institute for Basic Medical Science, School of Medicine, CHA University, Seongnam-si, Gyeonggi-do, Republic of Korea

**Keywords:** postpartum depression, microglia, P2RY12, IL-10, glial cells

## Abstract

Postpartum depression (PPD) is another type of depression, including emotional fluctuation, fatigue, and anxiety. Based on the specific event like giving birth, it can be speculated that PPD might have its specific mechanism. Here, we confirmed that dexamethasone (DEX) administration during pregnancy (gestational days 16–18) induced depressive- and anxiety-like behaviors in dam (DEX-dam) after weaning period (3 weeks). DEX-dam showed anxiety-like behaviors in open-field test (OFT) and light–dark test (LD). In addition, DEX-dam exhibited depressive-like behaviors with the increased immobility time in forced swimming test (TST). Molecular analysis confirmed that microglia, rather than neurons, astrocytes, and oligodendrocytes, are involved in anxiety-/depressive-like behaviors. Notably, *P2ry12*, homeostatic gene, and purinoceptor, along with hyper-ramified form, were reduced in the hippocampus of DEX-dam. In addition, we found that IL-10 mRNA was reduced in lymph nodes without alteration of pro-inflammatory cytokines, such as TNF-α, IL-1β, and IL-6. Interestingly, anxiety-/depressive-like behaviors of DEX-dam were restored with the normalization of *P2ry12* and *IL-10* after 10 weeks postpartum without antidepressants. Our results propose that stress hormone elevation during pregnancy might be associated with PPD via microglial P2RY12 and peripheral IL-10.

## 1. Introduction

Depression is a pluralistic and heterogeneous psychiatric disorder with multiple causes and incoherent responses to therapy (Belmaker and Agam, 2008; Malhi and Mann, 2018). The exact cause of depression is not fully understood; however, it is believed to be a combination of genetic, biological, environmental, and psychological factors (Otte et al., 2016). To mimic major depressive disorder in humans, there are methods to induce depressive- and anxiety-like behaviors in animals, including chronic stressful stimuli using restraint, unpredictable stress, or social defeat (Wang et al., 2017). Lipopolysaccharide (LPS) injection also induces depressive-like behaviors after sickness-like behaviors (O'connor et al., 2009). Chronic illnesses such as hypertension can also induce depressive-like behaviors in mice (Park et al., 2020). However, these methods have limitations in reflecting only some causative factors of major depressive disorder (MDD).

Postpartum depression (PPD) is one of the most common complications of parturition, with an estimated prevalence of ~ 20% (Wang et al., 2021). The pathophysiology of PPD is linked to neuronal circuit dysfunction, genetics, epigenetics, immune functions, and neuroendocrine and neurotransmitter imbalance (Stewart and Vigod, 2016). Based on the environmental risk factors of PPD (Misdrahi et al., 2005; Milgrom et al., 2008), stressful stimuli or glucocorticoid administration has been applied to pregnant mice as a PPD animal model (Mir et al., 2022). In addition, most mechanistic studies on PPD have focused on previously reported neuronal factors, although recent studies have reported the crucial role of the neuroimmune system and glial cells in MDD.

Microglia are central nervous system (CNS) phagocytes in the brain that play a crucial role in maintaining homeostasis. Recent research suggests that microglia may also play a role in the development of MDD (Rajkowska and Miguel-Hidalgo, 2007; Felger and Lotrich, 2013; Najjar et al., 2013). Most animal studies have shown that microglia have a reactive form in response to chronic stress, leading to neuroinflammation in the brain (Sugama et al., 2007; Tynan et al., 2010) although there are controversial results (Han et al., 2015) because microglia can also induce or resolve inflammation, maintain synaptic plasticity, and show phagocytosis in healthy and diseased brains, depending on the microenvironment (Colonna and Butovsky, 2017; You et al., 2023). Additionally, treatments that target microglia, such as anti-inflammatory drugs, can improve depressive- and anxiety-like symptoms in animal models (Bay-Richter and Wegener, 2022) although clinical trials have failed (Miller and Pariante, 2020). In human studies, translocator protein 18 kDa (TSPO), a protein found in the mitochondria of immune cells and some brain cells, including microglia, is increased, indicating that TSPO may contribute to the development of MDD (Meyer et al., 2020). In addition to microglia, other glial cells, such as astrocytes and oligodendrocytes, have also been reported to be related to neuropsychiatric disorders. Recent research has suggested that stress and other factors can alter the function of astrocytes and oligodendrocytes, leading to changes in brain function and behavior that are similar to those observed in MDD (Miguel-Hidalgo, 2022).

Peripheral immune system is involved in the pathogenesis of through communication with microglia in the CNS (Dantzer et al., 2008; Cherry et al., 2014; Miller and Raison, 2016; Takahashi et al., 2016; Himmerich et al., 2019). Numerous studies have reported that increased circulating pro-inflammatory cytokines, such as interleukin-1 beta (IL-1β), IL-6, tumor necrosis factor-alpha (TNF-α), and interferon-gamma (IFN-γ), are associated with MDD (Dowlati et al., 2010; Fagundes et al., 2013; Farooq et al., 2017) by activating indoleamine 2,3 dioxygenase (IDO). IDO breaks down tryptophan, a precursor of serotonin, into kynurenine (KYN) instead of serotonin, thereby reducing serotonin production (Lichtblau et al., 2013). KYN is converted to kynurenine acid by KAT (kynurenine aminotransferase) in astrocytes, which can cause cognitive deficits, and is transformed to quinolinic acid by KMO (kynurenine 3-monooxygenase) in microglia, which causes neurotoxicity, including oxidative stress and neurodegeneration (Brown et al., 2021). We previously reported that stress vulnerability could be overcome by peripheral IL-4 and IL-10, by restoring CX3CR1 decrease in microglia (Han et al., 2015).

Depression research has mainly focused on chronic stress stimuli, but depression has various pathological mechanisms. In this study, we investigated the pathological mechanism in postpartum depression induced by corticosterone-like drugs. Although many studies have focused on neurons in PPD (Maguire and Mody, 2008; Haim et al., 2014), a few studies have focused on glial cells. Previously, we reported that dexamethasone (DEX) administration during pregnancy induced schizophrenia-relevant behaviors in male offspring and depressive- and anxiety-like behaviors in female offspring (Rim et al., 2022). In this model, we examined depressive- and anxiety-like behaviors in postpartum dams following DEX administration during pregnancy. In addition, we attempted to identify a possible mechanism for PPD by focusing on glial cells.

## 2. Materials and methods

### 2.1. Experimental animals

Forty-three 13-week-old pregnant female C57BL/6N mice (Koatec Inc., Seoul, Korea) were used in this study. Information on which animals were used is presented in Table 1. Pregnant female mice were housed in cages that held three to five animals each, under specific pathogen-free conditions at 22 ± 0.5°C with a 12-h light–dark cycle and supplied with food and water *ad libitum* at the CHA BIO COMPLEX animal facility (Seongnam, Korea). After the weaning period, a series of behavioral assessments were performed on the dams. All behavioral assessments were conducted during the light phase of the cycle. Experimental procedures were approved by the Animal Care and Use Committee of CHA University (IACUC220088).

**Table 1:** Experimental process of animals.

	**Behavioral test**		**Dissection (WB, qPCR)**	**Perfusion (IHC)**	**Serum (ELISA)**
3 weeks (independent)	Control (*n =* 10) DEX (*n =* 12)	Control^*^ (*n =* 3) DEX^*^ (*n =* 7)	Control (*n* = 5) DEX (*n* = 7)	Control (*n* = 5) DEX (*n* = 5)	Control (*n* = 10) DEX (*n* = 10)
10 weeks (independent)	Control (n = 6) DEX (*n* = 6)		Control (*n* = 6) DEX (*n* = 6)	Control (*n* = 3) DEX (*n* = 7)	Control (*n* = 6) DEX (*n* = 6)

^*^Subsequent behavioral test and sampling at 10 weeks.

### 2.2. Dexamethasone treatment in pregnant mice

50 μg/kg DEX (Sigma, D1756) and physiological saline as control were injected subcutaneously into pregnant mice once a day for 3 days (gestational day: GD16 to 18) to recapitulate elevation of stress hormones during the antenatal period (Rim et al., 2022).

### 2.3. Behavioral test

Behavioral assessments were performed during the light cycle between 9:00 AM and 6:00 PM, with one assessment per day. Behavioral tests consisted of an open-field test (OFT), light and dark (LD) test, social interaction (SI) test, sucrose preference test (SPT), tail suspension test (TST), and forced swimming test (FST). All assessments were performed as blinded tests to minimize bias. Each behavioral assessment is described in Supplementary Data 1 and referred to our previous studies (Han et al., 2015; Park et al., 2020).

### 2.4. Enzyme-linked immunosorbent assay

Whole blood was collected by cardiac puncture when mice were sacrificed for perfusion and dissection. Serum was isolated by centrifugation at 448 g at 4°C for 20 min and stored at−80°C until assayed. Serum glucocorticoid, IL-1β, TNF-α, and IL-10 levels were measured using corticosterone ELISA kit (Enzo, ADI-901-097), IL-1β ELISA kit (R&D Systems, MLB00C), TNF-α ELISA kit (Enzo, ADI-900-047), and IL-10 ELISA kit (Abcam, ab255729), respectively, according to the manufacturer's instructions. Enzyme-reacted serum was read in a microplate reader at 450 nm except for corticosterone, and only corticosterone was read at 405 nm.

### 2.5. Total protein extraction and Western blot analysis

After behavioral assessments, Western blot was performed referring to our previous study (Rim et al., 2022). Briefly, the dissected hippocampi and amygdala were homogenized in radioimmunoprecipitation assay (RIPA) buffer (Thermo Fisher Scientific, 89900). The concentration of protein from the supernatant after centrifuge was measured using a protein assay reagent (Bio-Rad, #5000006) using bovine serum albumin as a standard. Protein lysates in sample buffer (Biosolution, BS0028) were incubated at 95°C for 10 min, separated by SDS-PAGE, and transferred onto polyvinylidene fluoride (PVDF) membranes. After blocking with 5% skim milk in Tris-buffered saline and 0.1% Tween-20 (TBST) for 1 h at room temperature, membranes were incubated with the primary antibodies, including anti-glucocorticoid receptor (GR) antibody (1:1,000, Santa Cruz, sc-1004), PSD95 antibody (1:500, Abcam, ab18258), synaptophysin antibody (1:10,000, Abcam, ab14692), BDNF antibody (1:1,000, Abcam, ab108319), pCREB antibody (1:500, Cell signaling, 9,198), CREB antibody (1:500, Cell signaling, 9,197), 5-HT1A receptor antibody (1:1,000, GeneTex, gtx104703), β-actin antibody (1:10,000, Cell signaling, 4,970), GAD67 antibody (1:500, Abcam, ab26116), CX3CR1 antibody (1:1,000, ProteinTech, 13885-1-AP), Arginase1 antibody (1:10,000, Novus, NB100-59740), myelin basic protein (MBP) antibody (1:10,000, Servicebio, GB12226), and glial fibrillary acidic protein (GFAP) antibody (1:10,000, Abcam, ab7260) at 4 °C overnight. Goat anti-rabbit IgG HRP-conjugated secondary antibody (1:10,000, Bethyl, A120-101P), Goat anti-mouse IgG HRP-conjugated secondary antibody (1:10,000, Bethyl, A90-116P), and Donkey anti-goat IgG HRP-conjugated secondary antibody (1:10,000, Bethyl, A50-101P) were incubated for 1 h at room temperature. The membranes were visualized using an ECL-plus solution (GE Healthcare, RPN2106). The membranes were exposed to chemiluminescence (LAS 4000, Fujifilm) to detect light emission. Quantification of band intensities was performed using the Fusion FX software (Vilber Lourmat).

### 2.6. Immunohistochemistry

Immunohistochemistry was performed referring to our previous study (Rim et al., 2022). Brain sections were incubated with the primary antibodies diluted with 1% NDS in PBS-T, including anti-c-Fos antibody (1:1,000, Abcam, ab190289), Iba-1 antibody (1:500, Wako, 019-19741), Olig2 antibody (1:300, Millipore, AB9610), MBP antibody (1:800, Servicebio, GB12226), and GFAP antibody (1:500, Abcam, ab7260) at 4 °C overnight. Brain sections were washed three times and incubated with Donkey anti-rabbit Alexa 488-conjugated secondary antibody (1:200, Invitrogen, A-21206), Donkey anti-mouse Alexa 488-conjugated secondary antibody (1:200, Invitrogen, A-21202), and Donkey anti-rabbit Alexa 647-conjugated secondary antibody (1:200, Invitrogen, A-31573) diluted with PBS-T at room temperature for 1 h. Brain sections were mounted using ProLong^TM^ Gold Antifade mounting solution with DAPI (Invitrogen, P36931). Fluorescent images were obtained using a confocal microscope (TCS SP5 II; Leica Microsystems, Wetzlar, Germany). One field of view (FOV) was obtained per brain region per mouse for confocal imaging. In 25 μm thickness brain section, images in 1 μm units were merged into a z-stack. All intensities in the region of interest (ROI) and counting of positive cells were analyzed using ImageJ software (National Institutes of Health, Bethesda, MD, USA). Dentate gyrus was taken from the level of interaural 1.98 mm, bregma−1.82 mm to the level of interaural 1.95 mm, bregma−1.85 mm. Amygdala was taken from the level of interaural 1.77 mm, bregma−2.03 mm to the level of interaural 1.74 mm, bregma−2.06 mm. External capsule was taken from the level of interaural 1.26 mm, bregma−2.54 mm to the level of interaural 1.23 mm, bregma−2.57 mm.

### 2.7. Fluorescence microscopy

To identify microglia and oligodendrocytes throughout the hippocampus, coronal brain sections were stained with Iba-1 and Olig2. To capture whole brain slides, automated slide scanning was performed using AxioScan Z1 slide scanner (Carl Zeiss Microscopy) at 20x magnification. In the middle 5 μm part of the 25 μm section, images in 1 μm units were merged into a z-stack; 405-nm and 488 nm lasers were used for the excitation of DAPI and AF488 fluorophores, respectively. Images were adjusted and obtained in Zen Blue image acquisition software (Zeiss). Counting of Iba-1- and Olig2-positive cells was analyzed with ImageJ. For counting without bias, the cells were counted by two blinded observers using ImageJ. Images by cell counting were analyzed by drawing each brain region with ImageJ to obtain unit area, and the counted cells were divided by each unit area.

### 2.8. Microglial cell morphometrics

The microglial morphology analysis was conducted according to our previous method (Yang et al., 2022). A total of 80–120 microglial cells per group (five mice, five fields per group) were used for skeletal analysis.

### 2.9. Quantitative polymerase chain reaction

The hippocampus, hypothalamus, amygdala, and mesenteric lymph nodes were analyzed for qPCR according to our previous study (Rim et al., 2022). Primer sequences are listed in Table 2.

**Table 2:** Primer sequences used for real-time qPCR.

**Primer**	**Forward (5′ → 3′)**	**Reverse (5′ → 3′)**
GAPDH	CGACCTTCACCATTTTGTCTACA	GCTTAAGAGACAGCCGCATCT
CSF1R	Qiagen #PPM03625F	
CX3CR1	TGGCCCAGCAAGCATAG	CATGTCTGCTACCCTCACAAA
P2RY12	TAACCATTGACCGATACCTGAAGA	TTCGCACCCAAAAGATTGC
TGF-b	TGACGTCACTGGAGTTGTACGG	GGTTCATGTCATGGATGGTGC
TREM2	TGGGACCTCTCCACCAGTT	GTGGTGTTGAGGGCTTGG
TNF-a	GAGTCCGGGCAGGTCTACTTT	CAGGTCACTGTCCCAGCATCT
IL-1b	GGCTGGACTGTTTCTAATGC	ATGGTTTCTTGTGACCCTGA
IL-4	ACAGGAGAAGGGACGCCAT	GAAGCCCTACAGACGAGCTCA
IL-6	AGTCCGGAGAGGAGACTTCA	ATTTCCACGATTTCCCAGAG
IL-10	GCAGCTCTAGGAGCATGTGG	ACAGCCGGGAAGACAATAACT
KMO	CCTGTAGAGGACAATATAGGATCAACAA	GCAAGCCCCATCTACTGCAT
IDO	GGCTTCTTCCTCGTCTCTCTATTG	TGACGCTCTACTGCACTGGATAC
KAT	CCAGGAACCCTTTATGCTATGAA	TGGAATAATCCCATGCTCATCA
C1qa	Qiagen #PPM24525E	
C3ar1	Qiagen #PPM04821A	
CD200R	AGGAGGATGAAATGCAGCCTTA	TGCCTCCACCTTAGTCACAGTATC
IGF-1	GCTTCGTTATCCACGACGATG	GAATGGCGGATCTTCACGTAG
Arg1	GGAATCTGCATGGGCAACCTGTGT	AGGGTCTACGTCTCGCAAGCCA
CD206	CTCTGTTCAGCTATTGGACGC	CGGAATTTCTGGGATTCAGCTTC
NOX2	GACCCAGATGCAGGAAAGGAA	TCATGGTGCACAGCAAAGTGAT
CRH	GTTAGCTCAGCAAGCTCACAG	GCCAAGCGCAACATTTCATTT
IL-17	CTCCAGAAGGCCCTCAGACTAC	GGGTCTTCATTGCGGTGG
IFN-γ	GGTCAACAACCCACAGGTCC	CAGCGACTCCTTTTCCGCTT
Tbx21	AGGGAACCGCTTATATGTCC	TCTCCATCATTCACCTCCAC
Gata3	AGAACCGGCCCCTTATCAA	AGTTCGCGCAGGATGTCC
Rorc	ACTACGGGGTTATCACCTGTGAG	GTGCAGGAGTAGGCCACATTAC
Foxp3	ACTCGCATGTTCGCCTACTTCAG	GGCGGATGGCATTCTTCCAGGT

### 2.10. Statistical analysis

The experimental data are presented as the mean ± error (SEM). The statistical significance of differences between groups was assessed with unpaired Student's *t*-tests using GraphPad Prism version 7 (GraphPad, La Jolla, CA, USA). Statistical significance was set at *P* < 0.05.

## 3. Results

### 3.1. DEX-treated postpartum dam exhibited anxiety- and depressive-like behaviors

**Figure 1:**
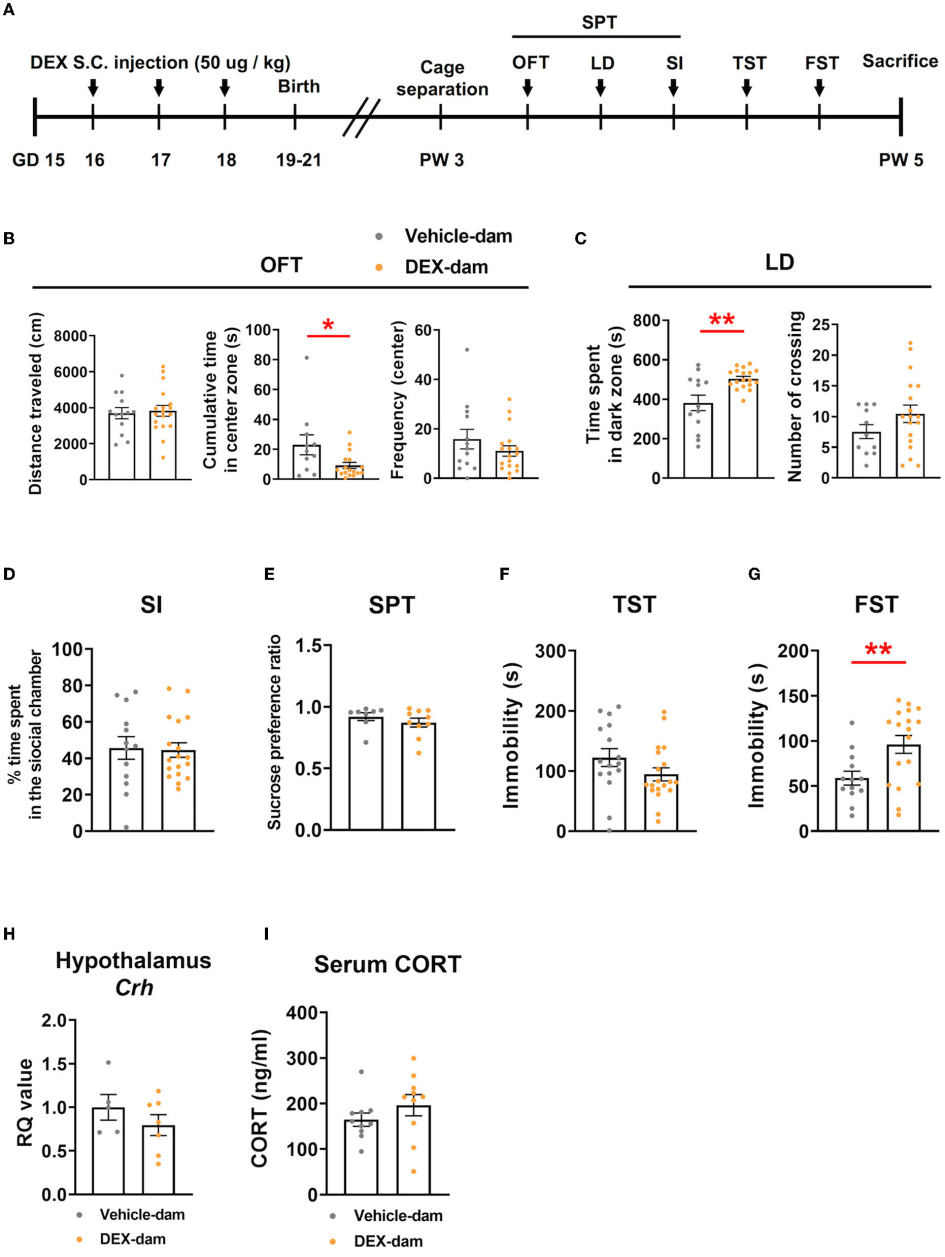
DEX-dam showed anxiety and depressive-like behaviors. **(A)** Pregnant mice were subcutaneously injected with dexamethasone (DEX, 50 μg/kg, once a day for 3 days) between gestational days 16 and 18, and we performed a series of behavioral assessments after weaning. DEX-dam showed anxiety-like behaviors. Less cumulative time in the center zone in an open-field test **(B**, OFT**)** and longer exploration in the dark zone in the light–dark box test **(C**, LD**)** compared with Vehicle-dam. DEX-dam did not show any changes in the social interaction test (**D**, SI), sucrose preference test **(E**, SPT**)**, and tail suspension test **(F**, TST**)** compared with Vehicle-dam. DEX-dam showed increased immobility time in the forced swimming test **(G**, FST**)** compared with Vehicle-dam. *n* = 13–18 in each group. **(H)** The expression level of CRH mRNA in the hypothalamus was by qRT-PCR. The RQ values are the ratio of the respective gene as a percentage of the controls. *n* = 5–7 in each group. **(I)** Serum corticosterone levels were measured by ELISA. *n* = 10 in each group. Data are presented as the mean ± standard error of the mean (SEM). For statistical analyses, we conducted an unpaired *t*-test; *p < 0.05, **p < 0.01, compared with Vehicle-dam.

To examine the effect of stress hormone elevation during pregnancy, we subcutaneously administered a synthetic glucocorticoid (dexamethasone; DEX, 50 μg/kg, GD16-18, once a day for 3 days) to pregnant mice. Behavioral assessments were performed in postpartum dams after 3 weeks, considering the weaning period. There was no difference in maternal care between the vehicle and DEX-dam groups. An overview of the experimental procedure is shown in Figure 1A. DEX-dam showed a decrease in the cumulative time of the center zone compared with the Vehicle-dam in the anxiety-related OFT (Figure 1B). DEX-dam spent a significantly longer time in the dark zone than the Vehicle-dam in the LD test (Figure 1C). In SI, which reflects sociality, there was no difference between the vehicle and DEX-dam (Figure 1D). In addition, there was no difference in the SPT, which reflects anhedonia, and sucrose consumption was not different (Figure 1E). The TST and FST were conducted to evaluate depressive-like behaviors. In the TST (Figure 1F), there was no difference between groups, but in the FST, DEX-dam showed a longer immobility time than Vehicle-dam (Figure 1G). After a series of behavioral tests, mice were sacrificed for molecular analysis. DEX-dams did not show hypothalamus–pituitary–adrenal axis (HPA axis) activation. The hypothalamic corticotropin-releasing hormone (CRH) mRNA expression and serum glucocorticoid (CORT) levels did not change in DEX-dam (Figures 1H, I).

### 3.2. Neuron-related factors were not changed in DEX-dam

**Figure 2:**
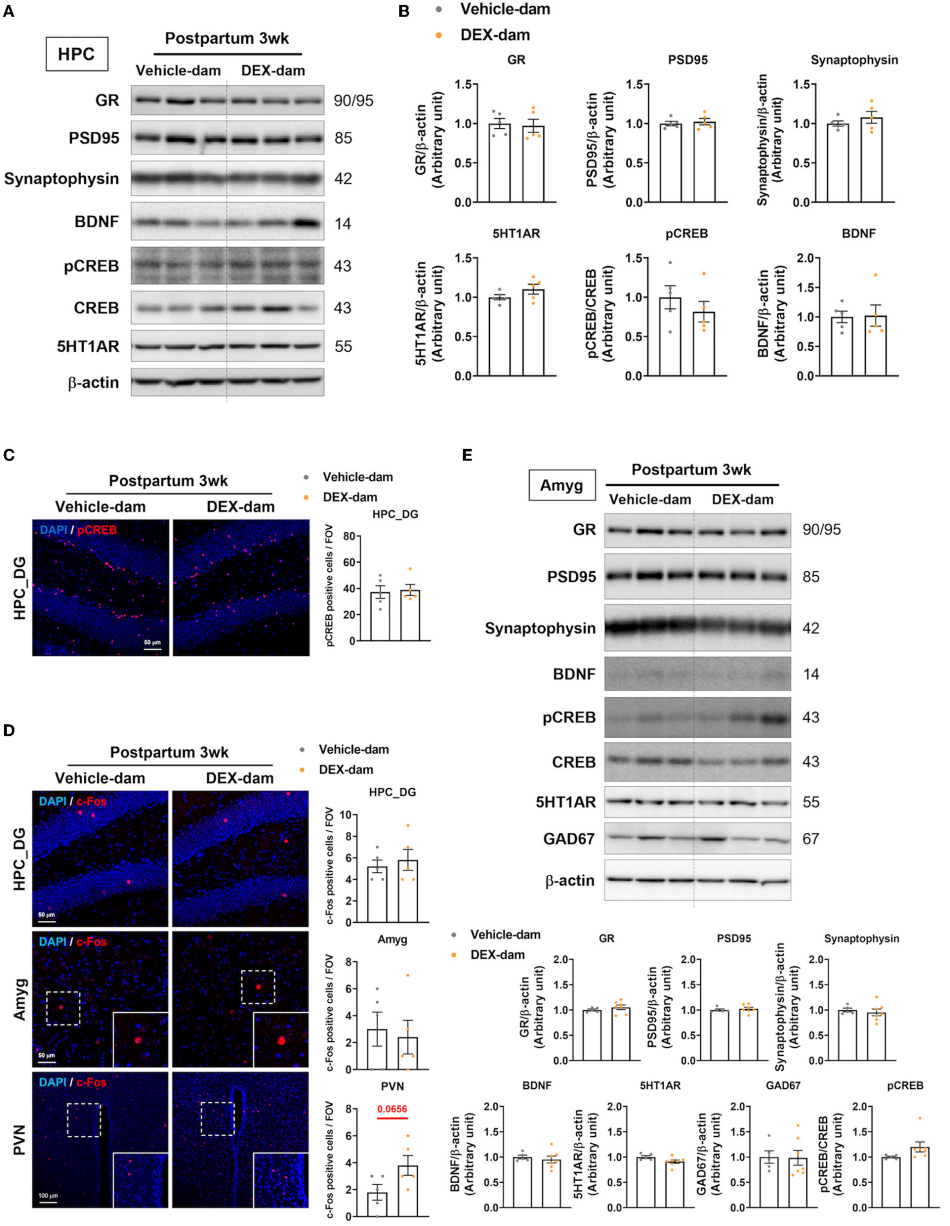
Change of anxiety and depression-related neuronal markers in the hippocampus. One day after the behavioral assessments (3 weeks postpartum), the mice were sacrificed for Western blot and immunofluorescence studies. **(A)** Western blot images of the glucocorticoid receptor (GR), postsynaptic density protein 95 (PSD95), synaptophysin, BDNF, pCREB, CREB, and 5-HT1AR in the hippocampus. **(B)** Relative quantification of Western blot images. n = 5 in each group. **(C)** Representative images of pCREB-positive cells in the dentate gyrus of the hippocampus (HPC_DG). pCREB (red) and DAPI (blue). Scale bar indicates (40x image, scale bar = 50 μm). *n* = 5 slices from five animal brains/group, one section per mouse. pCREB-positive cells were counted in the HPC_DG. **(D)** Representative images of c-Fos-positive cells in HPC_DG, amygdala (Amyg), and paraventricular nucleus (PVN) of the vehicle and DEX-dam groups. c-Fos-positive cells in PVN were counted in 40x images. c-Fos (red) and DAPI (blue). The white squares are magnified images of the dotted white square in Amyg, PVN. Scale bar indicates (HPC_DG, Amyg:40x image, scale bar = 50 μm/PVN:20x image, scale bar = 100 μm). *n* = 5 slices from 5 animal brains/group, one section per mouse. c-Fos-positive cells in the HPC_DG, Amyg, and PVN were counted. Quantification of c-Fos-positive cells in HPC_DG, Amyg, and PVN. **(E)** Western blot images of glutamic acid decarboxylase 67 (GAD67) Western blot images of the glucocorticoid receptor (GR), postsynaptic density protein 95 (PSD95), synaptophysin, BDNF, pCREB, CREB, and 5-HT1AR in the amygdala (Amyg) and the relative quantification of the Western blot images. *n* = 5–7 in each group. Data are presented as the mean ± standard error of the mean (SEM). For the statistical analysis, we conducted an unpaired *t*-test.

First, we examined well-known factors related to depressive- and anxiety-like behaviors in the hippocampus and amygdala. The protein levels of GR, postsynaptic density protein 95 (PSD95), synaptophysin, BDNF, pCREB, CREB, and 5-HT1AR were not changed in the hippocampus of DEX-dams (Figures 2A, B). In the immunofluorescence study, pCREB in the dentate gyrus of hippocampus was not altered, in line with the Western blot results (Figure 2C). To examine neuronal activity in the hippocampus dentate gyrus (HPC_DG), amygdala (Amyg), and paraventricular nucleus (PVN), c-Fos, a neuronal activity marker, immunoreactivity (IR) was stained, and the number of c-Fos-positive cells did not change between the two groups. However, neuronal activity tended to increase in the paraventricular nucleus (*p* = 0.0656) (Figure 2D). GABAergic neurons and synapses in the amygdala are known to be involved in regulating anxiety-related behaviors (Babaev et al., 2018). Thus, we examined the protein levels of glutamate decarboxylase 67 (GAD67), a GABAergic neurotransmission marker, using Western blotting. However, there was no difference in the levels of GAD67 as well as glucocorticoid receptor (GR), postsynaptic density protein 95 (PSD95), synaptophysin, BDNF, pCREB, CREB, and 5-HT1AR in the amygdala (Figure 2E).

### 3.3. Microglia are hyper-ramified with a reduction of *p2ry12* and *cd206* in DEX-dam

**Figure 3:**
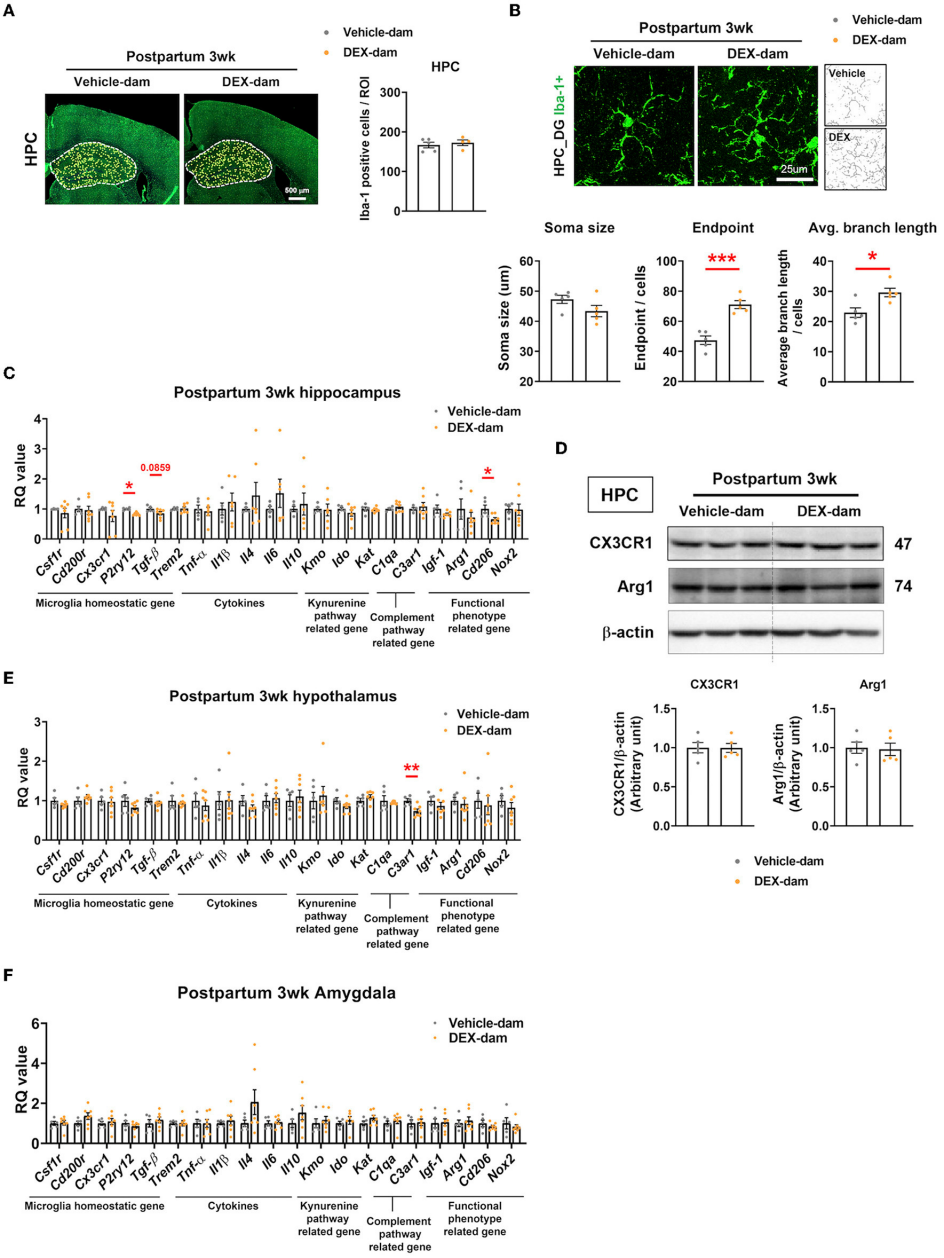
Microglia were hyper-ramified with reduction of *P2ry12* and *Cd206* in the hippocampus of DEX-dam. **(A)** Representative images of Iba-1-positive cells in the whole hippocampus region obtained with a slide scanner. Iba-1-positive cells were counted by ImageJ. Scale bar is indicated (20x image, scale bar = 500 μm). *n* = 4–5 slices from five animal brains/group. **(B)** Representative images of the morphology of Iba-1-positive cells from the dentate gyrus of the hippocampus (HPC_DG) obtained with a confocal microscope. Scale bar is indicated (40x image, scale bar = 25 μm). *n* = 5 in each group. The soma size, the number of endpoints, and the average branch length were analyzed in Iba-1-positive cells. **(C)** *P2ry12* and *Cd206* expression in the hippocampus of DEX-dam were decreased compared with Vehicle-dam. *n* = 4–7 in each group. **(D)** Protein levels of CX3CR1 and Arginase1 were evaluated by Western blot, and their expression levels were quantified by ImageJ. *n* = 5 in each group. **(E)** *C3ar1* expression in the hypothalamus of DEX-dam was decreased compared with Vehicle-dam. n = 5–7 in each group. **(F)** mRNA expression was not different between groups in the amygdala. *n* = 5–7 in each group. Data are presented as the mean ± standard error of the mean (SEM). For statistical analyses, we conducted an unpaired *t*-test, *p < 0.05, **p < 0.01, ***p < 0.001, compared with Veh-dam.

We investigated the number and morphology of microglia in the hippocampus of DEX-dam. There was no difference in the number of microglia (Iba-1-positive cells) in the hippocampus between the vehicle and DEX-dam groups (Figure 3A). Still, the average branch length and endpoints of DEX-dam were significantly increased in morphology analysis (Figure 3B), indicating a hyper-ramified form. Next, we examined the microglial functional phenotype-related factors. The mRNA levels of microglia homeostatic genes, cytokines, phagocytosis-related genes, and kynurenine pathway-related genes were investigated using qRT-PCR. In the hippocampus, *P2ry12* and C*d206* were lower in DEX-dams than in Vehicle dams, and *Tgf-*β showed a tendency to decrease (Figure 3C). The protein levels of CX3CR1 and Arginase1 (Arg1), which were related to stress vulnerability and depression in our previous studies, were not changed in the hippocampus (Figure 3D). In the hypothalamus and amygdala, which are regions related to depression and anxiety, there was no difference in microglia-related genes, except for *C3ar1* in the hypothalamus. C*3ar1* was decreased in DEX-dams (Figures 3E, F).

### 3.4. Oligodendrocytes and astrocytes were not involved in anxiety- and depressive-like behaviors in DEX-dam

**Figure 4:**
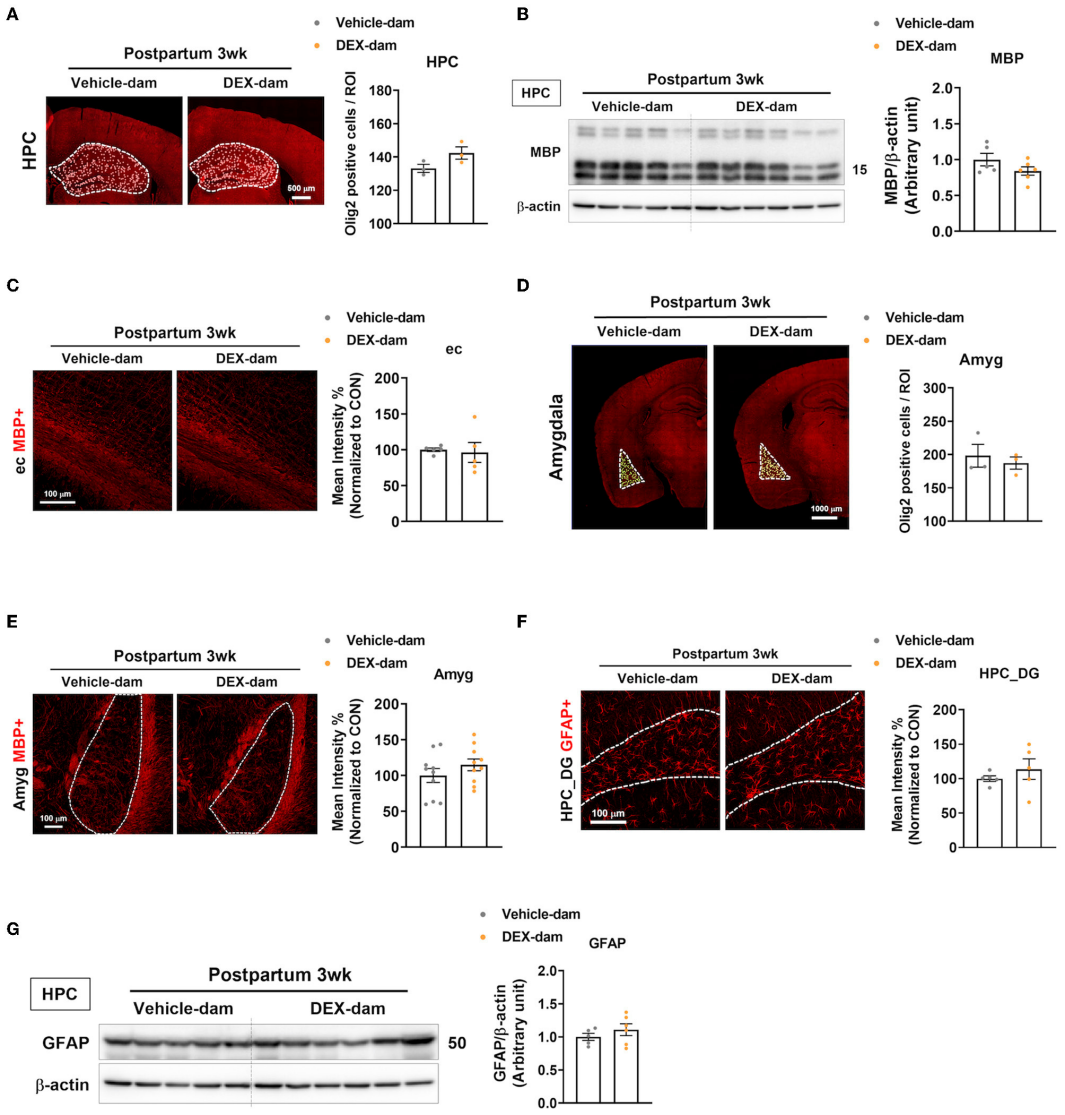
Oligodendrocytes and astrocytes were not changed in DEX-dam. **(A)** Representative images of oligodendrocyte transcription factor 2 (Olig2)-positive cells in the whole hippocampus region. Olig2-positive cells were counted by ImageJ. Scale bar is indicated (20x image, scale bar = 500 μm). *n* = 3 slices from three animal brains/group. **(B)** Western blot images of myelin basic protein (MBP) in the hippocampus. Relative density is defined as the ratio of respective protein density as a percentage of beta-actin density. n = 5–7 in each group. **(C)** Immunohistochemistry was performed to measure the intensity of MBP expression in the external capsule (ec) of vehicle and DEX-dam. Scale bar is indicated (40x image, scale bar = 100 μm). *n* = 5 slices from five animal brains/group, and intensity was measured in one section per mouse. **(D)** Representative images of Olig2-positive cells in the amygdala. Olig2-positive cells were counted by ImageJ. Scale bar is indicated (20x image, scale bar = 1000 μm). n = 3 slices from three animal brains/group. **(E)** Representative images of MBP expression in the amygdala (Amyg) of vehicle and DEX-dam. Scale bar is indicated (20x image, scale bar = 100 μm). *n* = 10 slices from five animal brains/group, and intensity was measured in two sections per mouse. **(F)** Immunohistochemistry was performed to measure the intensity of glial fibrillary acidic protein (GFAP)-positive cells in the hippocampus dentate gyrus (HPC_DG) of vehicle and DEX-dam. Scale bar is indicated (40x image, scale bar = 100 μm). *n* = 4–5 slices from five animal brains/group. **(G)** Protein levels of GFAP were measured by Western blot. Relative density is defined as the ratio of respective protein density as a percentage of beta-actin density. *n* = 5–7 in each group. Data are presented as the mean ± standard error of the mean (SEM). For statistical analyses, we conducted an unpaired *t*-test.

Along with the involvement of microglia in DEX-dams, oligodendrocytes and astrocytes were also analyzed. First, to examine the change in the number of oligodendrocytes in the hippocampus, immunofluorescence was performed with Olig2, an oligodendrocyte marker, and olig2-positive cells per ROI were counted using ImageJ. This showed that there was no difference compared with Vehicle-dam (Figure 4A). White matter abnormalities have been implicated in the pathogenesis of major depressive disorder (Tham et al., 2011; Cole et al., 2012; Zhou et al., 2021). Therefore, we conducted Western blotting to measure MBP, the most common cellular marker for myelin, in the hippocampus, but there was no difference (Figure 4B). Immunofluorescence studies also confirmed the results of Western blotting. MBP intensity was not altered in the external capsule (Figure 4C). As DEX-dam showed anxiety-like behaviors, oligodendrocytes were also analyzed in the amygdala, a brain region associated with anxiety. There was no change in the number of Olig2-positive cells and MBP intensity in the amygdala (Figures 4D, E). Next, we examined changes in astrocytes using GFAP as an astrocyte marker in the hippocampus. There was no difference in the intensity of GFAP in the hippocampus dentate gyrus (Figure 4F) between immunofluorescence and Western blots (Figure 4G). Taken together, except for microglia, other glial cells were not altered by DEX-dam.

### 3.5. DEX-dam exhibited reduced IL-10 in lymph nodes

**Figure 5:**
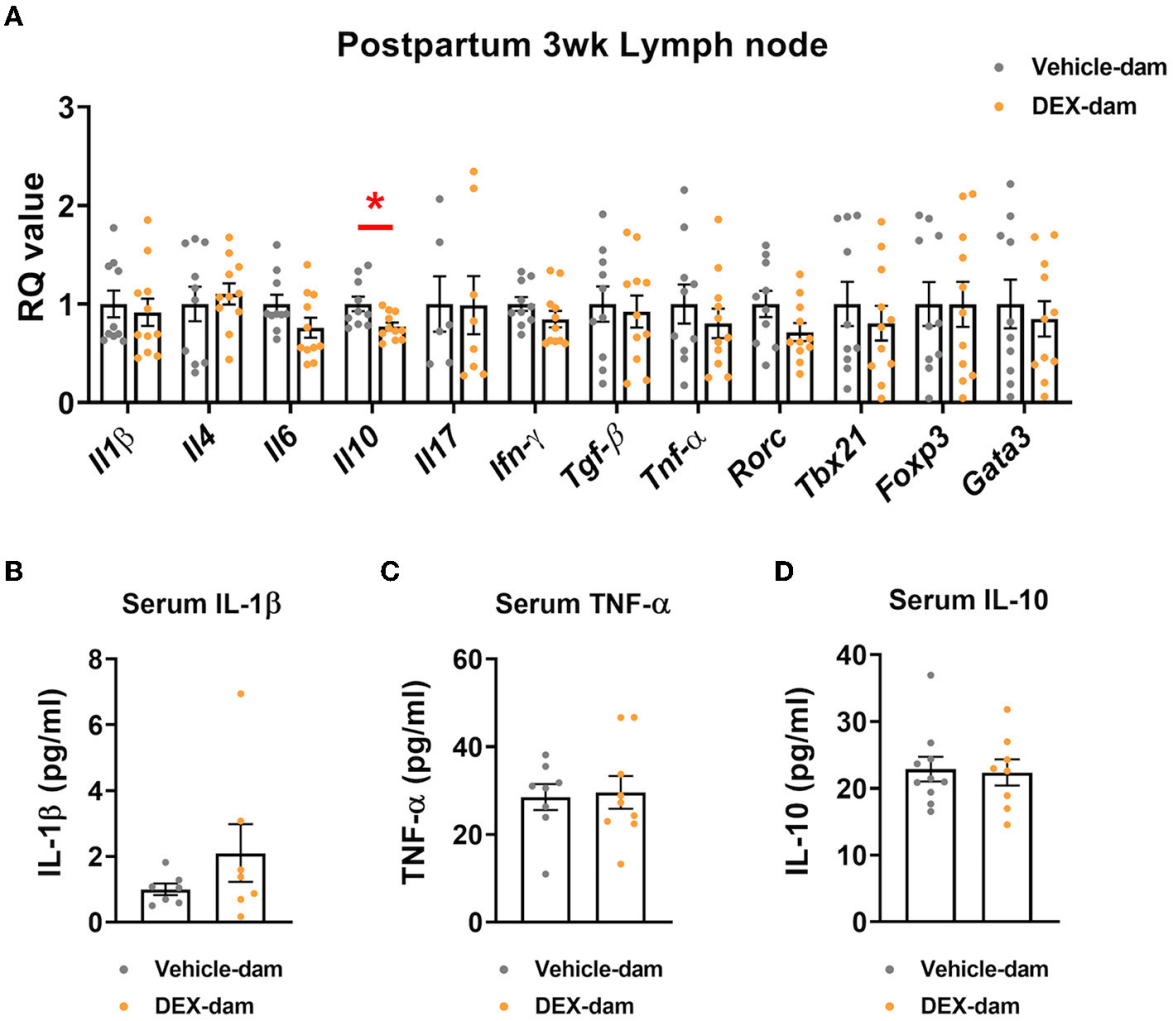
*Il-10* was decreased in the lymph node of DEX-dam. **(A)** Dissected mesenteric lymph nodes were analyzed by qRT-PCR. *n* = 10–11 in each group. RQ values represent the ratio of each transcription gene as a percentage of control. **(B–D)** Serum was obtained to measure circulating levels of pro-inflammatory cytokines, such as IL-1β and TNF-α, IL-10 using an ELISA kit, respectively. *n* = 7–10 in each group. Data are presented as the mean ± standard error of the mean (SEM). For statistical analyses, we conducted an unpaired *t*-test, *p < 0.05, compared with Veh-dam.

To examine the possible involvement of the peripheral immune system in anxiety-/depressive-like behaviors shown in DEX-dams (Han et al., 2015; Kohler et al., 2017), we measured the mRNA expression of cytokines in lymph nodes. We found that the mRNA expression levels of IL-10, an anti-inflammatory cytokine, were decreased in the mesenteric lymph nodes of DEX-dam. In contrast, other cytokines (e.g., IL-1β, IL-4, IL-6, IL-17, TGF-β, TNF-α) and T-cell subtype markers (e.g., Tbx21, Foxp3, gata3, RORγ) did not change (Figure 5A). Circulating IL-1β, TNF-α, and IL-10 levels in the serum did not change (Figures 5B–D).

### 3.6. Anxiety- and depressive-like behaviors in DEX-dam were alleviated without antidepressants at 10 weeks

**Figure 6:**
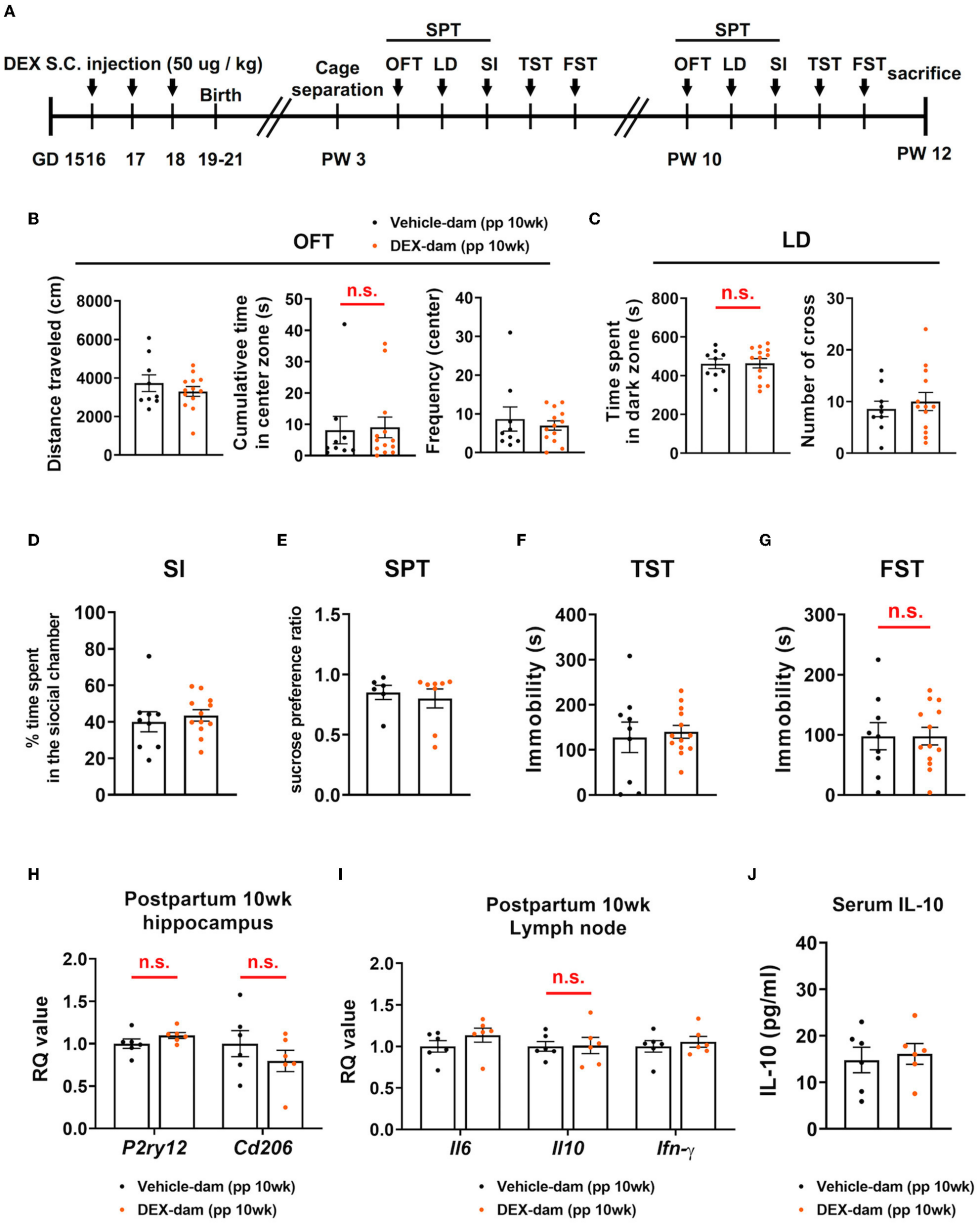
Anxiety- and depressive-like behaviors in DEX-dam were alleviated without antidepressant. **(A)** Experimental scheme for the behavioral test at 10 weeks postpartum (pp) of DEX-dam (pp 10wk DEX-dam). Anxiety-like behavior of DEX-dam shown in OFT was restored **(B)**, and longer exploration in the dark zone also disappeared in LD **(C)** compared with Vehicle-dam. DEX-dam did not show any difference in the SI **(D)**, SPT **(E)**, and TST **(F)** compared with Vehicle-dam. There is no difference in the immobility time of DEX-dam in the FST **(G)** compared with Vehicle-dam. *n* = 9 and *n* = 13 in each group. **(H)** The expression level of corticotropin-releasing hormone (CRH) mRNA in the hippocampus was measured by qRT-PCR. The RQ values are the ratio of the respective gene as a percentage of the control. *n* = 6 in each group. **(I)** Dissected mesenteric lymph nodes were analyzed by qRT-PCR. *n* = 6 in each group. **(J)** Serum was obtained to measure circulating levels of IL-10 using an ELISA kit. *n* = 6 in each group. Data are presented as mean ± standard error of the mean (SEM). For statistical analyses, we conducted an unpaired *t*-test.

A series of behavioral assessments were conducted at 10 weeks postpartum to determine whether anxiety-/depressive-like behaviors were maintained for a longer period in DEX-dam (Figure 6A). In the OFT, there was no difference in the cumulative time of the center zone compared with the Vehicle-dam, and the LD test showed no difference in time spent in the dark zone compared with the Vehicle-dam (Figures 6B, C). This finding suggests that anxiety-like behaviors were restored over time. In SI, there was no difference in DEX-dam compared with Vehicle-dam consistent with the 3-week results (Figure 6D). In addition, there was no difference between vehicle and DEX-dam in the SPT and TST (Figures 6E, F). In the FST, the immobility that increased in 3-week DEX-dams was recovered at 10 weeks (Figure 6G). The mRNA expression levels of *P2ry12* and *Cd206*, which decreased in the hippocampus at 3 weeks, were normalized at 10 weeks (Figure 6H). IL-6, IL-10, and IFN-γ mRNA levels were not different from those of the Vehicle-dam in the lymph nodes (Figure 6I). At 10 weeks, there was no difference in serum IL-10 level as at 3 weeks (Figure 6J).

## 4. Discussion

In the present study, we found that microglia may be involved in postpartum depression/anxiety, at least in our experimental design. First, we found that DEX injection during pregnancy induced postpartum depression- and anxiety-like behaviors in dams. Second, we investigated depression-/anxiety-related factors in neurons, microglia, astrocytes, and oligodendrocytes in DEX-dams and found that *P2ry12*, which is a purinoreceptor microglia-specific gene, decreased in the hippocampus. Third, peripheral IL-10, related to stress vulnerability, was also reduced in the lymph nodes. Interestingly, postpartum depressive- and anxiety-like behaviors in DEX-dams were restored without antidepressants over time. Although we cannot confirm whether DEX-dam represents postpartum depression/anxiety in humans, we propose the possible involvement of microglia and circulating IL-10 in postpartum depression/anxiety rather than neurons and other glial cells.

Studies have shown that chronic stress, a major risk factor for depression, can alter microglial morphology and function, leading to inflammation and oxidative stress in the brain (Tynan et al., 2010; Walker et al., 2013). This, in turn, contributes to the development of depression and anxiety. In addition, there is evidence that changes in microglial morphology can also be a result of depression, as depression has been shown to be associated with decreased microglial density and hyper-ramified patterns in the hippocampus. We also found that microglia have a hyper-ramified form with an increase in endpoint and branch length in DEX-dams. A long process and a number of endpoints might be so as to communicate more with neurons by extending toward neuronal synapses. Although changes in microglial morphology do not reflect their specific functional role and are not fully understood, hyper-ramified microglia indicate changes in homeostatic microglial function in DEX-dams.

Along with microglial morphological changes, we found that *P2ry12* was reduced in the hippocampus of DEX-dam. P2RY12 mediates microglial chemotaxis toward ADP/ATP gradients, leading to migration. A previous study demonstrated that P2RY12-knockout (KO) mice exhibited innate fear, leading to robust neuronal activity in the hippocampus when exposed to stressful situations (Peng et al., 2019). By extending microglial processes toward hyperactive neurons as sources of ATP, microglia might play a role in suppressing neuronal activity, similar to inhibitory neurons, leading to the protection of neurons from excessive activation (Badimon et al., 2020). The *P2ry12* reduction might be linked to the behaviors shown in DEX-dam, in association with the hyper-ramified morphology of microglia. Microglia extend their processes by sensing ATP levels. However, despite the extension process with multiple branches, microglia might have impaired ATP-P2RY12 downstream signaling due to the reduction of *P2ry12* in DEX-dams, although c-Fos IR was not changed in the hippocampus of DEX-dams.

We also found an *Il-10* reduction in lymph nodes. IL-10 released from microglia plays a crucial role in maintaining neuronal homeostasis, and IL-10 deficiency induces the inflammatory phenotype of microglia with an elevation of pro-inflammatory cytokines (Laffer et al., 2019). However, we found that DEX-dam did not show any change in IL-10 mRNA levels in the hippocampus. Peripheral IL-10 levels also affect the microglial function and homeostatic gene expression. Our previous study confirmed that intraperitoneal IL-10 administration restored reduced CX3CR1 levels in the hippocampus of mice with stress vulnerability (Han et al., 2015). In addition, intranasal IL-10 injection showed an antidepressant effect in mice with learned helplessness. IL-10 sensing is required to differentiate pathological microglia into homeostatic microglia (Shemer et al., 2020). P2RY12 is a microglial homeostatic gene and purinoreceptor. P2RY12 was decreased in the spinal cord of mutant SOD1 mice that mimicked amyotrophic lateral sclerosis pathology, although Iba-1-positive microglia were increased, indicating that the loss of microglia homeostatic genes might be involved in microglial dysfunction in neurodegenerative diseases (Butovsky et al., 2015). Thus, *P2ry12* reduction in DEX-dams might be associated with failure to return to homeostatic microglia, which might be involved in IL-10 reduction. However, further studies are required to confirm this speculation.

We did not find any evidence related to the involvement of other glial cells in DEX-dam. Astrocytes, oligodendrocytes, and microglia express GR. Among them, GR mRNA levels in microglia are greater than those of both mineralocorticoid receptors and estrogen receptors, suggesting that microglia are more sensitive to DEX exposure (Sierra et al., 2008). The relative abundance of GRs in microglia may explain why microglia respond uniquely to DEX exposure during pregnancy. We have also reported that DEX induces hyper-ramified dysfunctional microglia with a reduction in homeostatic microglial genes, including *P2ry12* (Park et al., 2019). The pyramidal layer of the hippocampus expresses high levels of GR. Chronic stress induced neuronal damage in the hippocampal CA3 region. However, depression-/anxiety-related proteins that were mainly expressed in neurons did not change in DEX-dam. We administered DEX for 3 days to induce postpartum depression/anxiety. Thus, relatively short-term DEX exposure does not seem to be sufficient to induce neuronal damage and change markers observed in other depression/anxiety animal models.

DEX-dam showed a differential pathomechanism compared with other animal models with anxiety/depression. Chronic stress using restraint, unpredictable, or social defeat commonly leads to impaired neurogenesis with BDNF reduction in the hippocampus (Lindholm and Castren, 2014). HPA activation and GR reduction in the hippocampus are hallmarks of depression. Neuroinflammation is also involved in treatment-resistant depression. In our study, DEX-dam did not show any changes related to previously reported depression-/anxiety-related proteins in the hippocampus, amygdala, or hypothalamus. Although morphological changes were observed in microglia, inflammatory cytokines, such as TNF-α, IL-1, and IL-6, were not increased in the hippocampus. Nevertheless, DEX-dam showed depressive-/anxiety-like behaviors. However, the behaviors were not maintained until 10 weeks postpartum, indicating restoration without antidepressants over time. These results indicate that our model, which mimics postpartum depression, might represent a part of heterogeneous depression/anxiety, especially among various PPD clinical phenotypes.

In conclusion, we explored the well-known depression/anxiety factors in the periphery, as well as neurons and glial cells of DEX-dams showing depressive-/anxiety-like behaviors. We found that the microglial response to DEX among CNS cells and IL-10 mRNA levels were reduced in lymph nodes. Although we cannot confirm the link between IL-10 and P2RY12 in PPD observed in DEX-dams, we propose that these two factors might be involved in our model.

## Data availability statement

The original contributions presented in the study are included in the article/Supplementary material, further inquiries can be directed to the corresponding author.

## Ethics statement

The animal study was reviewed and approved by the Animal Care and Use Committee of the CHA University (IACUC220088).

## Author contributions

H-JK wrote the manuscript and conducted behavioral and molecular experiments. M-JY and SS conducted the behavioral study. CR analyzed the behavioral and molecular data. M-SK supervised all processes, determined the direction of the manuscript, and approved the final submission of the manuscript. All authors critically revised and reviewed the manuscript.
