# Supplementary material for: Possible involvement of microglial P2RY12 and peripheral IL-10 in postpartum depression
Source: Front Cell Neurosci. 2023 Jun 15;17:1162966. doi: 10.3389/fncel.2023.1162966 (PMC10309556; doi:10.3389/fncel.2023.1162966)
Supplement: Supplementary file 1 [file Data_Sheet_1.docx]

**Behavioral test**

(a) Open field test (OFT)

The open field test (OFT) was performed to measure locomotor activity and exploratory behaviors. The apparatus consisted of a white Plexiglas® box (50 × 50 × 40 cm). After the acclimation period, mice were placed in the center and allowed to explore the box freely for 10 minutes. The total distance moved and the time spent in the center and four corner zones were recorded and measured using EthoVision XT9 video tracking system. After each test, the apparatus was cleaned with 70% ethanol solution.

(b) Light and dark (LD)

Anxiety-like behavior was assessed through LD by identifying their tendency to avoid bright places. The apparatus consists of a box (30 × 30 × 30 cm) connected to an open bright area and a closed dark area. The areas were connected by a small square hole (7 × 7 cm). Mice were placed in the center of the bright area, then the number of transitions between the areas and time spent in the dark area were manually measured for 10 minutes.

(c) Social interaction (SI)

The social interaction box is a rectangular, three-chambered box made of white Plexiglas® (60 × 40 × 40 cm). Each chamber was 20 × 40 × 40 cm. Walls separated by clear plexiglass have small openings (square, 6 cm×6 cm) to allow access to each chamber. Mice were able to move between the chambers through small side opening in the compartments. Plastic cylinders in each of the 2 side chambers contained probe mice or a dummy, and the test and probe mice were able to contact each other through a lot of 1 × 1 cm holes in the cylinders. A test mouse was placed in the center of the chamber and allowed to acclimate for 2 minutes. During the adjustment period, the entrance connecting each chamber was blocked. After 2 minutes of adaptation period, an unfamiliar same sex probe mouse with no previous contact with the subject mouse was placed in one of the two confinement cylinders in the side chamber. The dummy was placed in the rest of restraining cylinders. Time spent in each of the 3 chambers was measured using the EthoVision tracking system for 5 minutes, and social preference was defined as (time spent in the social chamber/total time) × 100%.

(d) Sucrose preference test (SPT)

To assess anhedonia, it was measured whether the mice preferred sucrose solution over drinking water. Mice were provided with two bottles, one filled with 1% sucrose diluted in drinking water and one filled with drinking water alone. Mice were acclimated to the two bottle conditions for two consecutive days and their sucrose preference was measured for additional two days. The position of the bottles was interchanged every day during 4 days of testing to avoid bias. Sucrose preference was quantified as (sucrose intake/total intake) × 100% on each test day.

(e) Tail suspension test (TST)

The tail suspension test (TST) was performed to measure immobility demonstrating depressive-like behavior. Mice with tape wrapped around their tails were suspended on a device with a hook attached to the top of a cupboard. Then, a hole was made in the tape at the end of the tail and carefully hung 2 cm away from the top of the hook. The data of the mice that climbed on their tails were excluded from analysis. The immobility time was measured during a 7 minutes of test period.

(f) Forced swimming test (FST)

The forced swimming test (FST) was used to evaluate the ability of mice to cope with an inescapable stressful situation, which reflects depressive-like behavior. Mice were individually placed in a 2 L Pyrex beaker (13 cm diameter, 24 cm height), filled with 23°C water with a depth of 17 cm. All mice were forced to swim for 6 minutes, and the duration of immobility was measured during the last 5 minutes of the test. The immobility of mice was considered as floating in water with no additional movement other than slightly moving to keep their head above the water.
